# Effect of Media Use on Adolescent Body Weight

**DOI:** 10.5888/pcd15.180206

**Published:** 2018-11-21

**Authors:** Eun Me Cha, Deanna M. Hoelscher, Nalini Ranjit, Baojiang Chen, Kelley Pettee Gabriel, Steven Kelder, Debra L. Saxton

**Affiliations:** 1 Michael & Susan Dell Center for Healthy Living, University of Texas Health Science Center at Houston, School of Public Health in Austin, Austin, Texas; 2 Department of Epidemiology, Human Genetics, and Environmental Sciences, University of Texas Health Science Center at Houston, School of Public Health in Austin, Austin, Texas; 3 Department of Biostatistics, University of Texas Health Science Center at Houston, School of Public Health in Austin, Austin, Texas; 4 Maternal and Child Health Epidemiology, Community Health Improvement Division, Texas Department of State Health Services, Austin, Texas

## Abstract

**Introduction:**

Adolescents spend a substantial amount of time consuming media, including watching television, playing video games, and using electronic devices to access the internet. We examined the relationship between prolonged media use on screen devices and its potential association with obesity through several mechanisms.

**Methods:**

We used data from 659,288 eighth and eleventh grade students who participated in the 2015–2016 School Physical Activity and Nutrition (SPAN) survey in Texas to examine the associations between hours of media use per day and 3 behaviors related to obesity: timing of last food intake, unhealthy eating behavior, and sleep hours. Also, mediation analyses were conducted to examine the pathways between hours of media use and body mass index (BMI).

**Results:**

Compared with adolescents who used media 2 hours or less per day, those who used media 6 hours or more had higher odds of nighttime eating (odds ratio [OR], 3.16; 95% confidence interval [CI], 1.76–5.66) and inadequate sleep (OR, 1.57; 95% CI, 1.05–2.36) and a higher coefficient for Unhealthy Eating Index score (3.87; 95% CI, 1.3–6.37). Mediation analysis demonstrated that for males sleep hours and timing of last food intake mediated the pathway between hours of media use and BMI. For females, unhealthy eating behavior mediated this pathway.

**Conclusion:**

Adolescents who used electronic media 6 or more hours at night had higher odds of unhealthy eating behavior and inadequate sleep hours than those with 2 hours’ use or less. Attention to behaviors associated with adolescents’ prolonged media use is needed to reduce risk of obesity.

## Introduction

Adolescents are inundated with media and spend more than 6 hours each day watching television, YouTube, and movies; playing video games; listening to music; and surfing the internet (1). Use of television and other screen devices (eg, smartphone, tablets, computers) is associated with risk of obesity through a variety of mechanisms, including insufficient physical activity and increased calorie intake while using screen devices (2,3).

Several studies have shown that increased media use is associated with shorter and poorer quality sleep (3,4), which is also a significant risk factor for obesity (5,6). After-school screen time is associated with increased size of evening snack portions and overall poor diet quality in adolescents (7). Moreover, epidemiologic studies have reported that consuming most daily calories in the evening is associated with higher body mass index (BMI) and an increased risk of obesity and metabolic syndrome. Taken together, media use is associated with negative effects on a variety of adolescent health behaviors, including unhealthy eating at night and inadequate sleep hours, which can ultimately lead to increased risk of overweight and obesity (2–9). However, few studies have examined the association between media use and timing of last food intake, unhealthy eating, and inadequate sleep hours in a representative sample of adolescents. Because Texas has the second largest population of US states and is racially diverse (10), patterns observed there may be used as an indicator of national prevalence of media use and related behaviors among adolescents.

The two objectives of our study were 1) to examine the association between categories of increased hours of media use as the targeted exposure variable and 3 behavioral outcomes (timing of last food intake, unhealthy eating behaviors, and hours of sleep, stratified by sex); and 2) to test the mediation effects of timing of last food intake, unhealthy eating behavior, and sleep hours between hours of media use and BMI, stratified by sex. We hypothesized that media use would be positively associated with the 3 behavioral outcomes and that these outcomes would also act as mediators between hours of media use and BMI in an adolescent population. This article was written in accordance with the STrengthening the Reporting of OBservational Studies in Epidemiology (STROBE) statement (11).

## Methods

**Study design and sampling**. Data were obtained from the School Physical Activity and Nutrition Survey (SPAN), a surveillance system designed to identify factors among school-age children that may underlie obesity, including dietary behaviors, nutrition knowledge and attitudes, and physical activity (12). Since 2000, SPAN has collected these serial cross-sectional data over 4 time periods (2000–2002, 2004–2005, 2009–2011, and 2015–2016). SPAN’s stratified, multistage probability sampling scheme yielded samples that represent 8th and 11th grade students in Texas. Further details on SPAN sampling are presented elsewhere (13). In our study, all 8th and 11th grade student respondents from the most recent 2015–2016 SPAN data were included (weighted count, 659,288; unweighted count, 9,056; 52.7% 8th graders and 47.3% 11th graders). Students with missing data (4.7%, n = 423) were excluded from the analyses.

### Data collection

Trained field staff members administered the SPAN questionnaire and obtained anthropometric data at randomly selected schools. The questionnaire included items about demographic information, diet, and physical activity. The validity of food frequency questionnaire items was evaluated with 24-hour food recall, and reliability of the questionnaire was established with test–retest methods (14). The SPAN protocol was approved by The University of Texas Health Science Center’s Committee for Protection of Human Subjects (HSC-SPH-17–0965).

### Measures

**Hours of media use**. Hours of media use were measured by asking how many hours per day the student usually watched or used 1) television, 2) a computer for schoolwork, 3) a computer for outside schoolwork, and 4) video games. For each of the 4, the 8 ordinal responses for per-day use were 0 (I don’t use or watch [specific media]), 0.5 (I watch less than 1 hour), 1 (1 hour), 2 (2 hours), 3 (3 hours), 4 (4 hours), 5 (5 hours), and 6 (6 hours or more per day). Hours of use of the 4 media were summed as a continuous variable ranging from 0 to 24 and stratified into 2 hours or less, 3 to 5 hours, and 6 hours or more per day. The questions about television viewing and video gaming were tested previously for their reliability with test–retest κ value 0.71 and 84% agreement (14); computer questions were adapted from the television and gaming questions.

**Timing of last food intake**. The timing of last food intake was measured with the question, “What is the latest time that you usually eat or drink anything (except water)?” on school days and on weekends. The response categories were before 7 PM, 7 PM to 7:59 PM, 8 PM to 8:59 PM, 9 PM to 9:59 PM, 10 PM to 10:59 PM, 11 PM to 11:59 PM, and 12 AM or later. These were collapsed into 3 categories: before 7 PM, between 7 PM and 10 PM, and 10 PM or later. We used 7 PM and 10 PM as the earliest and latest cutoffs on the basis of the average dinner time for adults (15) and the definition of nighttime eating (16). The responses were combined by taking an average of the recorded responses for weekdays and weekend days.

**SPAN unhealthy eating index**. We created a SPAN unhealthy eating index, which was based on methods used in previous work (17), as a summary measure of unhealthy food items, identified as fried meats, sugary drinks, salty fried snacks, and various desserts. Frequency measures of the consumption of each food item were added and scaled to a range of 0 to 100 — the higher the value, the unhealthier the diet. Healthy foods were not included in the analyses, but foods such as baked meat, vegetables, fruits, milk, yogurt, and whole-grain pasta and bread were assessed by additional SPAN survey questions.

**Sleep**. SPAN measured hours of sleep with a single question: “On an average school night, how many hours of sleep do you get?” with 7 response options: 4 or less, 5, 6, 7, 8, 9, or 10 or more. This construct, adopted from the Youth Risk Behavior Surveillance System (YRBSS) questionnaire (18), was treated both as a continuous variable and a categorical variable (<8 h, 8–9 h, and ≥10 h).

**Bodyweight**. The SPAN field staff measured students’ height to the nearest 0.1 cm and weight to the nearest 0.1 kg on site. Measurements were taken with shoes and socks off with a digital scale (Tanita BWB-800S) and a stadiometer (Perspective Enterprise Portable Adult/Infant Measuring Unit PE-AIM-101). Interrater reliability was assessed for a 5% sample of the population and showed a strong agreement. BMI percentile and weight status were determined by using the Centers for Disease Control and Prevention (CDC) standard growth charts for children and adolescents: healthy weight (<85th percentile), overweight (85th percentile to <95th percentile), and obese (≥95th percentile) (19).

**Covariates**. Demographic variables were age, grade (8th or 11th), sex (male or female), and race/ethnicity (white/other, Hispanic, black), which were used in the multistage probability sampling scheme. School-level poverty status was estimated by the proportion of students who were eligible for free or reduced-price lunch at each school (20) and were categorized into tertiles where the highest poverty status was represented by the upper tertile. Physical activity was measured by asking, “During the past 7 days, on how many days were you physically active for a total of at least 60 minutes per day?” Answers were stratified into those who were active for 7 days or less than 7 days during the past week.

### Statistical analysis

All analyses were performed by using SAS 9.4 (SAS Institute, Inc) where complex multistage survey design and sampling weights were accounted by using PROC SURVEY procedures. Descriptive statistics examined the distribution of hours of media use, timing of last food intake, sleep hours, and unhealthy eating behavior, stratified by sex. A Rao-Scott χ^2^ test was conducted to evaluate the difference between the sexes. Three separate weighted regression analyses were performed to examine separately the associations between categories reflecting hours of media use and the 3 outcomes (unhealthy eating behaviors, timing of last food intake, and hours of sleep). A linear regression model was conducted for unhealthy eating behaviors, and multinomial logistic regression models were conducted for timing of last food intake and sleep hours. Models Analyses for each of the 3 outcomes were stratified by sex and adjusted for age, race/ethnicity, physical activity, and economic disadvantage tertile (model 1). Furthermore, additional dependent variables (timing of last food intake, unhealthy eating behavior, and hours of sleep) were added to produce the full model (model 2).

We conducted mediation analyses to separate the dynamic relationship between hours of media use and BMI percentile via timing of last food intake, unhealthy eating behaviors, and hours of sleep (21). All mediation analyses were stratified by sex and were implemented in Mplus Version 7 (Muthén & Muthén).

## Results

Most of our sample of 659,288 adolescents were Hispanic/Latino (50.9%), and 59.8% had a healthy BMI (<85^th^ percentile) (Table 1). Overall, 37.2% of adolescents reported nighttime eating (at 10 PM or later), with higher proportions of girls (39.4%) than boys (35.1%; *P* = .30). The percentage of nighttime eating was 20% greater on weekends than on weekdays. With regard to sleep, 58.8% of our sample reported sleeping less than 8 hours per day (62.5% of girls and 55.3% of boys) (*P* = .001) (Table 1).

**Table 1:** Demographic Characteristics of Respondents, Study of Effect of Media Use on Body Weight Among Adolescents, Texas 2015–2016^a^^,^ b

Variable	All	Boys	Girls	*P* Value^c^
**Unweighted sample size, no. **	9,056	4,555	4,501	NA
**Weighted sample size, no.**	659,288	336,613	322,675	
**Sex, % (CI)**	NA	51.1 (46.9–55.2)	48.9 (44.8–53.1)	.60
**School grade**				
8th	52.7 (39.9–65.6)	53.1 (39.3–67.0)	52.3 (39.1–65.5)	.90
11th	47.3 (34.4–60.1)	46.9 (33.0–60.7)	47.7 (34.5–60.9)	
**Age, mean (SD)**	15.0 (0.2)	15.0 (0.2)	14.9 (0.2)	.80
**Race/ethnicity**				
Black	12.5 (8.0–17.1)	12.5 (7.8–17.2)	12.5 (7.4–17.7)	>.99
Hispanic	50.9 (43.1–58.8)	50.9 (42.0–59.7)	51.0 (42.8–59.2)	
White/other	36.6 (28.1–45.0)	36.6 (26.8–46.4)	36.5 (28.2–44.8)	
**Economic disadvantage tertiles^d^**				
Lowest	46.5 (34.0–59.0)	47.9 (34.3–61.4)	45.1 (32.5–57.7)	.60
Middle	29.6 (20.2–39.1)	29.7 (19.1–40.3)	29.5 (20.0–39.1)	
Upper	23.9 (12.1–35.6)	22.4 (11.3–33.6)	25.4 (12.5–38.2)	
**Body mass index^e^**				
Healthy weight	59.8 (56.8–62.8)	57.7 (52.6–62.9)	62.0 (58.5–65.4)	.01
Overweight	17.9 (15.8–20.0)	16.7 (13.1–20.2)	19.2 (16.7–21.7)	
Obese	22.3 (19.3–25.2)	25.6 (21.3–29.9)	18.8 (16.4–21.2)	
**Timing of last food intake, mean of weekdays and weekends**				
Before 7 PM	12.3 (8.7–15.8)	13.4 (7.7–19.1)	11.2 (8.3–14.0)	.30
Between 7 PM and 10 PM	50.5 (47.1–53.9)	51.5 (46.5–56.5)	49.4 (45.3–53.5)	
10 PM or later	37.2 (34.2–40.3)	35.1 (31.1–39.1)	39.4 (36.0–42.9)	
**Timing of last food intake, weekdays only**				
Before 7 PM	17.3 (13.7–20.9)	18.3 (12.4–24.2)	16.2 (13.3–19.2)	.04
Between 7 PM and 10 PM	50.1 (46.3–53.9)	53.0 (48.0–58.0)	47.2 (42.6–51.7)	
10 PM or later	32.6 (29.9–35.3)	28.7 (25.3–32.1)	36.6 (32.9–40.3)	
**Timing of last food intake, weekends only**				
Before 7 PM	11.1 (7.9–14.3)	13.3 (7.6–19.1)	8.8 (6.8–10.9)	.03
7 PM–10 PM	35.9 (32.6–39.1)	37.5 (32.5–42.5)	34.2 (30.8–37.5)	
10 PM or later	53.0 (49.7–56.3)	49.2 (44.7–53.7)	57.0 (53.3–60.7)	
**Hours of sleep, mean (SD)**	7.0 (0.2)	7.1 (0.2)	6.9 (0.2)	.91
<8	58.8 (54.7–63.0)	55.3 (49.9–60.7)	62.5 (58.7–66.4)	.001
8–9	37.8 (34.0–41.5)	41.9 (36.8–47.0)	33.5 (30.1–36.8)	
≥10	3.4 (2.2–4.5)	2.8 (1.5–4.1)	4.0 (2.4–5.6)	
**Hours of media use**				
≤2	11.3 (9.6–13.0)	9.7 (7.6–11.8)	13.0 (10.9–15.0)	.10
3–5	26.2 (22.5–29.9)	28.3 (22.6–34.0)	24.0 (20.0–28.1)	
≥6	62.5 (58.4–66.6)	62.0 (55.9–68.2)	63.0 (58.7–67.3)	
**Unhealthy eating behavior, tertile, mean (SD)**	50.9 (0.2)	50.4 (0.3)	51.4 (0.2)	.90
Lowest	33.0 (29.7–36.4)	35.3 (31.5–39.1)	30.6 (26.6–34.7)	.006
Middle	41.1 (36.5–45.7)	42.1 (37.1–47.1)	40.1 (35.0–45.2)	
Upper	25.9 (23.1–28.6)	22.6 (18.8–26.3)	29.3 (25.9–32.6)	
**Reported days of physical activity**				
≥7	22.5 (20.5–24.4)	31.3 (27.5–35.0)	13.4 (10.9–15.9)	<.001
<7	77.5 (75.6–79.5)	68.7 (65.0–72.5)	86.6 (84.1–89.1)	

Abbreviations: CI, confidence interval; NA, not applicable; SD, standard deviation.

a Data are from the School Physical Activity and Nutrition Survey, 2015–2016 (32).

b Values are weighted percentage (95% CI) unless otherwise indicated.

c Rao-Scott χ^2^ test was used to calculated *P* values.

d Economic status data were obtained from the Texas Education Agency (20).

e CDC standard growth charts for children and adolescents. (https://www.cdc.gov/healthyweight/bmi/calculator.html) were used to classify BMI categories (healthy weight, <85th percentile; overweight, 85th–95th percentile; obese, ≥95th percentile).

Among all adolescents in our sample, the SPAN unhealthy eating index increased by 3.87 units (95% CI, 1.38–6.37) for those who used media 6 hours or more per day compared with those who used media 2 hours or less per day (Table 2). This overall association also remained significant in model 2. However, when stratified by sex, only the association for girls in model 1 remained significant (coefficient 3.03; 95% CI, 1.55–4.51).

**Table 2:** Weighted Regression Models, Unhealthy Eating Behavior, Study of Effect of Media Use on Body Weight Among Adolescents^a^, Texas 2015–2016^b^

Hours of Media Use	Coefficient (95% Confidence Interval)		
	All	Boys	Girls
** Model 1^c^**			
≤2	0	0	0
3–5	0.28 (−2.19 to 2.74)	−0.63 (−2.43 to 1.17)	0.21 (−1.04 to 1.45)
≥6	3.87 (1.38 to 6.37)	1.22 (−0.15 to 2.58)	3.03 (1.55 to 4.51)
**Model 2^d^**			
≤2	0	0	0
3–5	−0.41 (−3.00 to 2.17)	−0.79 (−2.51 to 0.93)	−0.20 (−1.32 to 0.93)
≥6	2.73 (0.45 to 5.01)	0.93 (−0.40 to 2.26)	2.35 (0.95 to 3.75)

a Weighted number, 659,288; unweighted number, 9,056.

b Data are from the School Physical Activity and Nutrition (SPAN) Survey, 2015–2016 (32).

c Model 1: Adjusted for age, race/ethnicity, physical activity, and economic disadvantage tertiles.

d Model 2: Adjusted for variables in model 1 plus timing of last food intake and sleep hours.

For timing of last food intake, the odds of nighttime eating (eating last food at 10 PM or later relative to 7 PM or earlier) were 3.16 (95% CI, 1.76–5.66) times higher for adolescents who used media 6 hours or more per day than those who reported 2 hours or less of media use (Table 3). These positive associations for nighttime eating were significant in both sexes.

**Table 3:** Weighted Regression Models, Timing of Last Food Intake^a^, Study of Effect of Media Use on Body Weight Among Adolescents^b^, Texas 2015–2016^c^

Hours of Media Use	Odds Ratio (95% CI)								
	All			Boys			Girls		
	Before 7 PM	7 PM–10 PM	10 PM or later	Before 7 PM	7 PM–10 PM	10 PM or later	Before 7 PM	7 PM–10 PM	10 PM or later
**Model 1^d^**									
≤2	1 [Reference]								
3–5	1 [Reference]	1.84 (1.14–2.96)	2.16 (1.25–3.70)	1 [Reference]	1.96 (1.07–3.60)	2.73 (1.25–5.96)	1 [Reference]	1.77 (0.76–4.12)	1.92 (0.70–5.28)
≥ 6	1 [Reference]	1.32 (0.74–2.37)	3.16 (1.76–5.66)	1.00^e^	1.34 (0.68–2.61)	3.50 (1.61–7.61)	1 [Reference]	1.44 (0.66–3.13)	3.33 (1.66–6.66)
**Model 2^e^**									
≤2	1 [Reference]								
3–5	1 [Reference]	1.92 (1.11–3.31)	2.20 (1.27–4.15)	1 [Reference]	2.01 (1.05–3.81)	2.76 (1.26–6.02)	1 [Reference]	1.79 (0.82–3.89)	1.89 (0.73–4.93)
≥ 6	1 [Reference]	1.29 (0.70–2.39)	2.66 (1.51–4.69)	1 [Reference]	1.44 (0.64–3.22)	3.03 (1.40–7.78)	1 [Reference]	1.38 (0.68–2.84)	2.78 (1.52–5.10)

a Weighted number, 659,288; unweighted number, 9,056.

b Mean of weekdays and weekends.

c Data are from the School Physical Activity and Nutrition (SPAN) survey, 2015–2016 (32).

d Model 1: Adjusted for age, race/ethnicity, physical activity, and economic disadvantage tertiles.

e Model 2: Adjusted for variables in model 1 plus unhealthy eating behavior and sleep hours.

**Figure 1:**
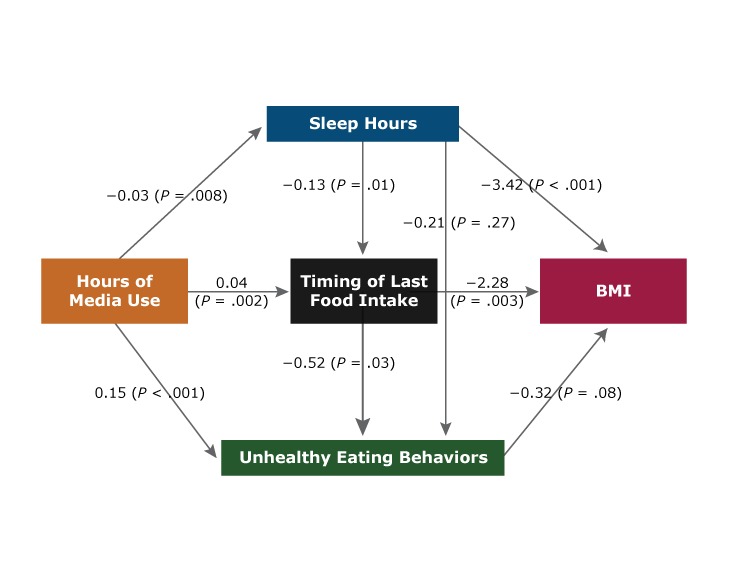
Mediation model examining the association between hours of media use and body mass index (BMI) percentile among adolescent males (8th and 11th grade students) in Texas, 2015–2016. Data are from the 2015–2016 School Physical Activity and Nutrition Survey (32).

**Figure 2:**
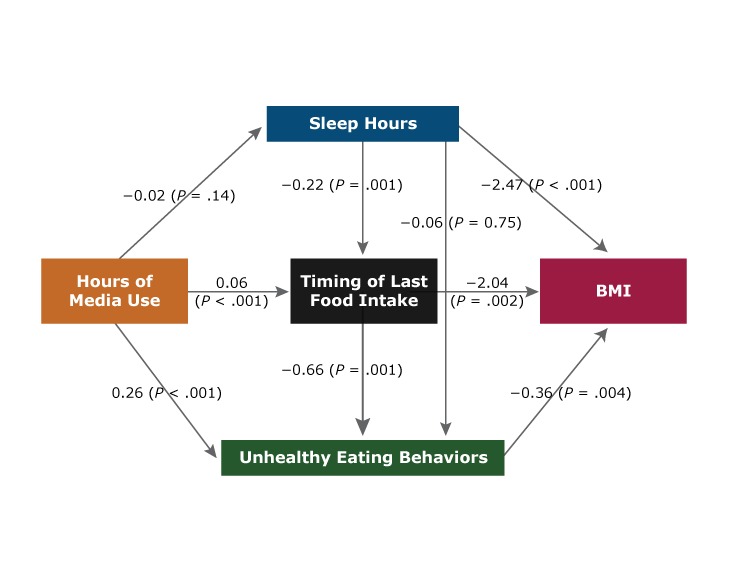
Mediation model examining the association between hours of media use with body mass index (BMI) percentile among adolescent girls (8th and 11th grade students) in Texas, 2015–2016. Data are from the 2015–2016 School Physical Activity and Nutrition Survey (32).

The odds of sleeping less than 8 hours per day relative to 8 to 9 hours were 1.57 (95% CI, 1.05–2.36) times higher for adolescents who used media more than 6 hours per day compared with those who used media 2 hours or less (Table 4). This association remained significant for boys only. Among boys, the only significant mediation effect (β = 0.017, *P* = .008) between hours of media use and BMI percentile (Table 5) was that of sleep hours. This relationship was also reflected in direct paths from hours of media use to sleep hours (β = −0.03, *P* = .008) and from sleep hours to BMI percentile (β = −3.42, *P* < .001) (Figure 1). The indirect effect of hours of media use via timing of last food intake was also positively associated with unhealthy food intake (β = 0.015, *P* = .05) (Table 5) among boys, with a strong direct pathway from timing of last food intake to unhealthy eating behavior (β = 0.52, *P* = .03) (Figure 1). Among teenage girls, negative mediation effects of timing of last food intake (β = −0.019, *P* = .02) and unhealthy eating behavior (β = −0.016, *P* = .009) were observed between hours of media use and BMI percentile (Table 5). The timing of last food intake and unhealthy eating behavior together acted as a mediator (β = −0.002, *P* = .03) between media use and BMI percentile. Hours of media use were negatively associated with BMI percentile via timing of last food intake and unhealthy eating behavior among girls (Table 5). This relationship was also reflected in direct pathways from timing of last food intake to BMI percentile (β = −2.04, *P* = .002) and from unhealthy eating behaviors to BMI percentile (β = −0.36, *P* = .004) (Figure 2). However, hours of media use were positively associated with unhealthy eating behavior via timing of last food intake (β = 0.024, *P* = .002), which means that as hours of media use increased, timing of last food intake played a significant role in increasing unhealthy food intake (Table 5).

**Table 4:** Weighted Regression Models, Hours of Sleep, Study of Effect of Media Use on the Timing of Last Food Intake and Body Weight Among Adolescents^a^, Texas 2015–2016^b^

Hours of Media Use	Hours, Odds Ratio (95% Confidence Interval)								
	All			Boys			Girls		
	<8	8–9	≥10	<8	8–9	≥10	<8	8–9	≥10
**Model 1^c^**									
≤2	1 [Reference]								
3–5	1.32 (0.86–2.02)	1 [Reference]	0.42 (0.20–0.90)	1.49 (0.85–2.61)	1 [Reference]	0.55 (0.12–2.60)	1.17 (0.74–1.86)	1 [Reference]	0.33 (0.09–1.24)
≥6	1.57 (1.05–2.36)	1 [Reference]	0.51 (0.22–1.14)	1.90 (1.27–2.84)	1 [Reference]	0.57 (0.23–1.46)	1.30 (0.77–2.21)	1 [Reference]	0.48 (0.14–1.61)
**Model 2^d^**									
≤2	1 [Reference]								
3–5	1.29 (0.82–2.03)	1 [Reference]	0.41 (0.21–0.81)	1.42 (0.77–2.62)	1 [Reference]	0.44 (0.13–1.54)	1.16 (0.73–1.84)	1 [Reference]	0.37 (0.11–1.18)
≥ 6	1.44 (0.93–2.24)	1 [Reference]	0.55 (0.28–1.09)	1.73 (1.11–2.67)	1 [Reference]	0.56 (0.22–1.42)	1.18 (0.67–2.10)	1 [Reference]	0.53 (0.20–1.43)

a Weighted number, 659,288; unweighted number, 9,056.

b Data are from the School Physical Activity and Nutrition Survey, 2015–2016 (32).

c Model 1: Adjusted for age, race/ethnicity, physical activity, and economic disadvantage tertiles.

d Model 2: Adjusted for variables in model 1 plus unhealthy eating behavior and timing of last food intake.

**Table 5:** Indirect Paths in Mediation Model, by Sex^a^, Study of Effect of Media Use on Body Weight Among Adolescents^b^, Texas 2015–2016^c^

Hours of Media Use	Body Mass Index Percentile, β (*P*)				Unhealthy Eating Behavior, β (*P*)
	(via) Timing of Last Food Intake	(via) Unhealthy Eating Behavior	(via) Sleep Hours	(via) Timing of Last Food Intake and Unhealthy Eating Behavior	(via) Timing of Last Food Intake
Boys	— d	— d	0.017 (.008)	— d	0.015 (.05)
Girls	−0.019 (.02)	−0.016 (.009)	— d	−0.002 (.03)	0.024 (.002)

a Weighted number, 659,288; unweighted number, 9,056.

b All results are weighted and adjusted for age, race/ethnicity, physical activity, and economic disadvantage tertiles.

c Data are from the School Physical Activity and Nutrition Survey, 2015–2016 (32).

d Not significant.

## Discussion

We evaluated the distribution of hours of media use and its association with timing of last food intake, sleep hours, and unhealthy eating behavior in a representative sample of 8th and 11th grade Texas adolescents. According to the Kaiser Family Foundation, children and adolescents aged 8 to 18 years spend an average of 7.5 hours per day using media, which totals 114 full days of media use in a year (22). In our study population, 88.7% of adolescents reported media use for 3 or more hours per day, which was higher than the percentage in the 2017 YRBSS report for 3 hours or more per day of television viewing (20.7%) and video games or computer use (43.0%) (3).

Overall, hours of media use were positively associated with unhealthy eating behaviors, nighttime eating, and inadequate sleep. The association between hours of media use and increased consumption of fast foods, snacks, and sugary drinks in adolescents was well established in previous studies (23,24). Moreover, media use is known to be a distracting activity that suppresses sensations of satiety and fullness when eating (25). Eating is often accompanied by media use, especially at night. Media use can also mimic the gratifying aspects of food as a way to mitigate negative emotions (26).

Hours of sleep are another concern. A growing body of literature indicates that the prevalence of inadequate sleep (<8 hours per night) was high among adolescents who used media devices (3,27). These results were also confirmed in our sample; the odds of having inadequate sleep were higher for those who used media more than 6 hours per day than those who used it 2 hours or less. In a systematic review, Gradisar suggested several mechanisms by which media use may affect sleep duration and quality: 1) media use may shorten sleep hours; 2) media use before sleep may trigger emotional, mental, or physiological alertness; and 3) light emission from the screen may interfere with sleep (28).

In our study, sleep hours were inversely associated with BMI percentile for both sexes. Accumulating evidence from laboratory and epidemiologic studies supports the premise that inadequate sleep duration and poor sleep quality are risk factors for develoPMent of obesity (5,6). Spiegel and colleagues showed alterations of hormone levels in healthy young men (ie, decrease in leptin levels and increase in ghrelin levels) and increased reports of hunger and appetite after sleep restriction (5). Moreover, mediation analyses revealed that sleep hours functioned as a significant mediator between hours of media use and BMI percentile in boys. In girls, hours of media use were negatively associated with BMI percentile via timing of last food intake and unhealthy eating behavior. This result was contrary to previous studies where a significant longitudinal correspondence between time spent on media use and increased body fat was observed from childhood to adolescence (29). However, in cross-sectional studies, the results were somewhat mixed: some reported no evidence of a significant association between use of media and BMI (24,29), and others reported that media use appeared to be positively associated with BMI for girls only (29,30). Taken together, these inconsistent results further suggest the need for more prospective longitudinal studies to evaluate the effect of media use, timing of last food intake, sleep hours, and unhealthy eating behavior on BMI.

Our study has strengths and limitations. SPAN was a cross-sectional survey with multistage probability sampling, which enabled us to generalize the results to Texas adolescents. Because of the sampling procedure, its results cannot be extended to other adolescent populations. However, because the SPAN sample is racially/ethnically diverse, the patterns observed in Texas may be used to forecast future national trends in adolescents. Nonetheless, temporality of exposure and outcome cannot be determined because of the cross-sectional study design. Hours of media use include computer use for schoolwork and represent overall media use rather than media use for leisure alone. Although we took the anthropometric measurements in our study, all other variables were self-reported and therefore subject to recall and social desirability bias. Previous studies have shown that people who are overweight or obese tend to overreport socially accepted behavior (ie, eating healthier or less food) than those with normal bodyweight (31). To measure eating behavior precisely, portion size and frequency of eating would need to be assessed.

Our study extended earlier work by investigating pathways between media use and BMI in an adolescent population. Results indicated that long hours of media use were associated with unhealthy eating behavior at nighttime; thus, the incidence of overweight and obesity may escalate in the near future in adolescents who engage in excessive media use. Therefore, it is crucial to evaluate interventions that focus on decreasing adolescents’ media use to prevent overweight and obesity and other related chronic health conditions. Strategies to decrease media use can include parental limits and school-wide guidelines for appropriate media use.
